# Supplementary material for: ClpG Provides Increased Heat Resistance by Acting as Superior Disaggregase
Source: Biomolecules. 2019 Dec 2;9(12):815. doi: 10.3390/biom9120815 (PMC6995612; doi:10.3390/biom9120815)
Supplement: Supplementary file 1 [file biomolecules-09-00815-s001.pdf]

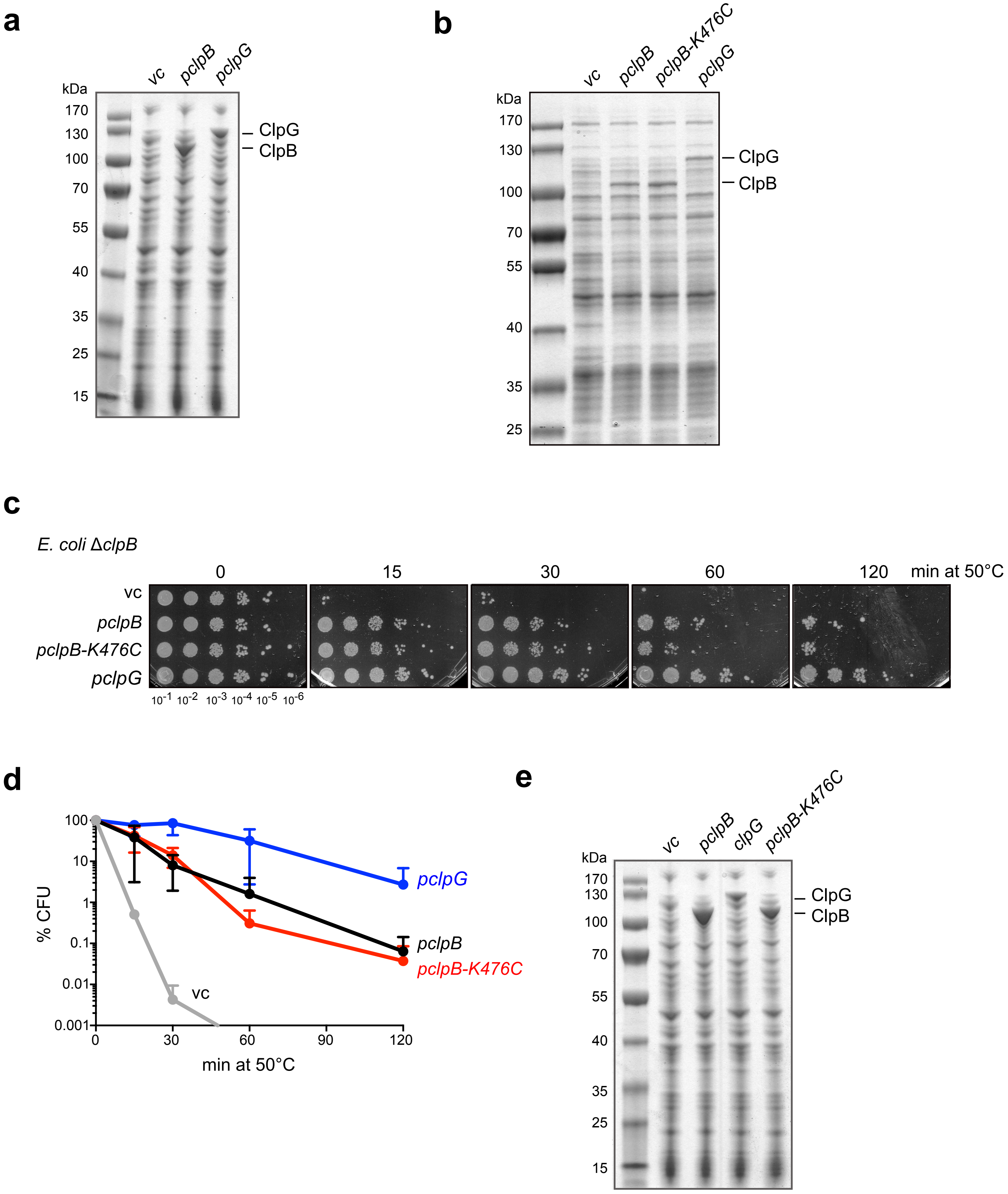
a
kDa
vc
pclpB
pclpG
170
130
100
70
55
40
35
25
15
ClpG
ClpB
b
kDa
vc
pclpB
pclpB-K476C
pclpG
170
130
100
70
55
40
35
25
ClpG
ClpB
c
E. coli ΔclpB
0
15
30
60
120
min at 50°C
vc
pclpB
pclpB-K476C
pclpG
10-1 10-2 10-3 10-4 10-5 10-6
d
% CFU
100
10
1
0.1
0.01
0.001
0
30
60
90
120
min at 50°C
pclpG
pclpB
pclpB-K476C
vc
e
kDa
vc
pclpB
clpG
pclpB-K476C
170
130
100
70
55
40
35
25
15
ClpG
ClpB

Figure S1

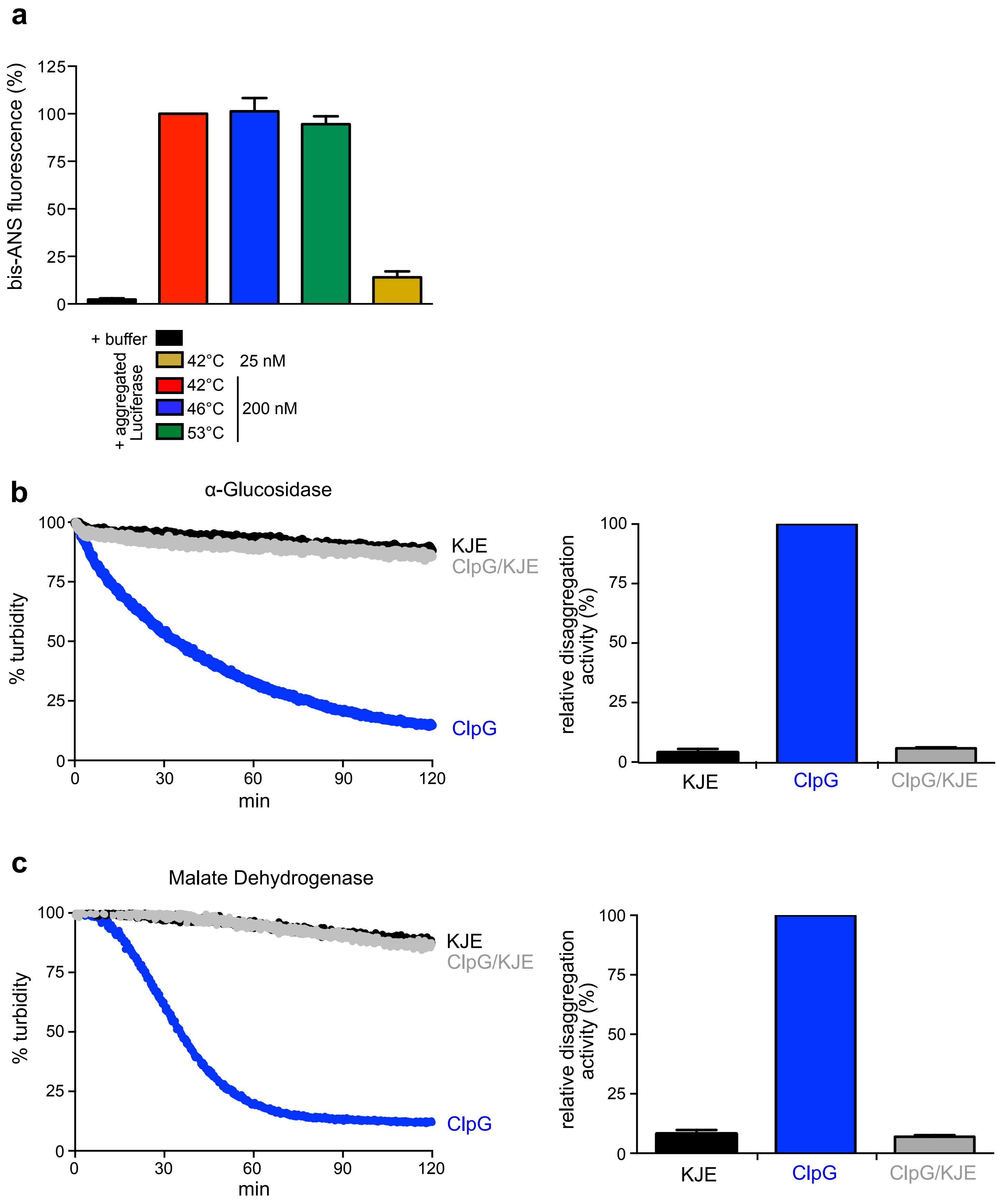
a
bis-ANS fluorescence (%)
125
100
75
50
25
0
+ buffer
+ aggregated Luciferase
42°C 25 nM
42°C
46°C
53°C
200 nM
b
α-Glucosidase
% turbidity
min
KJE
ClpG/KJE
ClpG
relative disaggregation activity (%)
KJE
ClpG
ClpG/KJE
c
Malate Dehydrogenase
% turbidity
min
KJE
ClpG/KJE
ClpG
relative disaggregation activity (%)
KJE
ClpG
ClpG/KJE

Figure S2

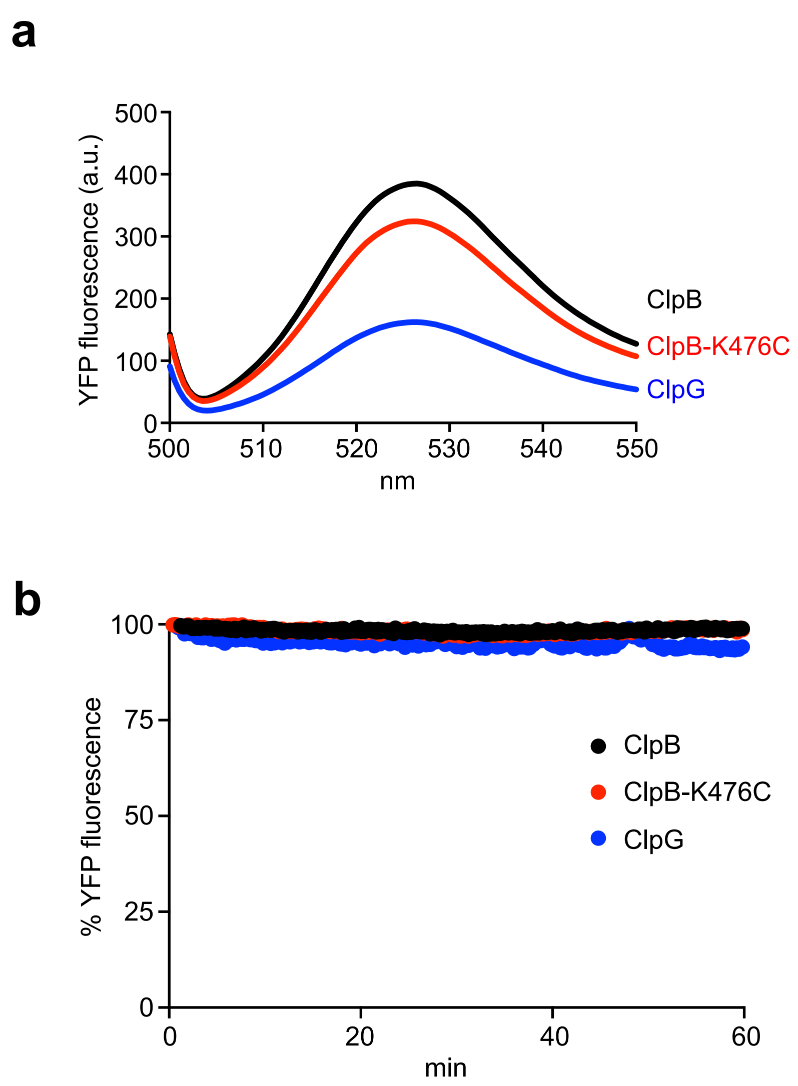
a
500
400
300
200
100
0
YFP fluorescence (a.u.)
500
510
520
530
540
550
nm
ClpB
ClpB-K476C
ClpG
b
100
75
50
25
0
% YFP fluorescence
0
20
40
60
min
ClpB
ClpB-K476C
ClpG

Figure S3

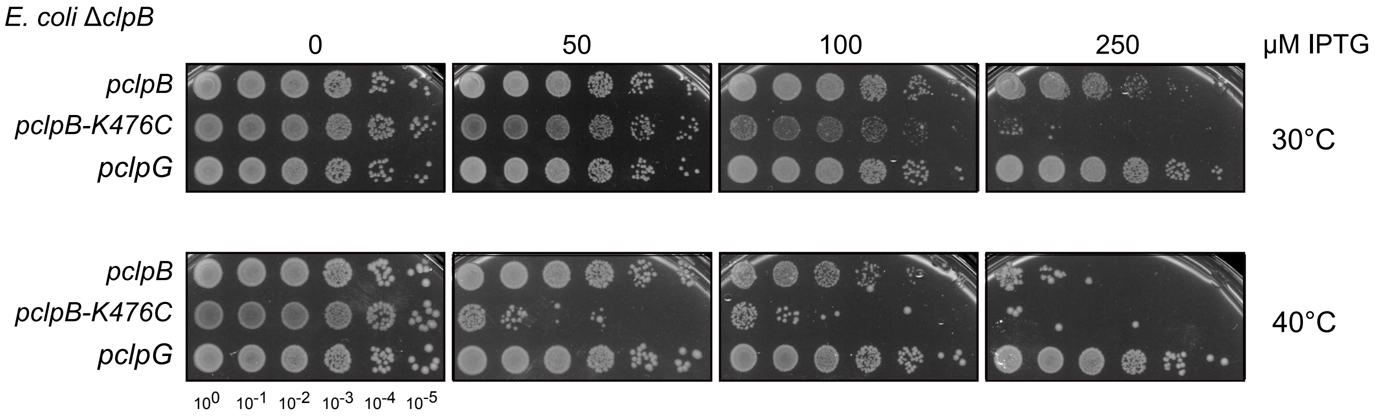
E. coli ΔclpB
0
50
100
250
µM IPTG
pclpB
pclpB-K476C
pclpG
30°C
pclpB
pclpB-K476C
pclpG
40°C
10^0 10^-1 10^-2 10^-3 10^-4 10^-5

Figure S4

Supplementary figure legends

Figure S1

Expression of *clpB* and *clpG* alleles in *E. coli ΔclpB* cells. (a) *E. coli ΔclpB* cells harboring plasmids for expression of *E. coli clpB* or *P. aeruginosa clpG* (vc: empty vector control) were grown at 30°C to mid-logarithmic growth phase. Expression of *clpB* and *clpG* alleles was induced by IPTG addition. Total cell lysates were prepared and analyzed by SDS-PAGE and Coomassie staining. The positions of ClpB and ClpG are indicated. A protein marker is given. (b) *E. coli ΔclpB* cells harboring plasmids for constitutive expression of Luciferase and IPTG-controlled expression of *E. coli clpB, E. coli clpB-K476C* or *P. aeruginosa clpG* (vc: empty vector control) were grown at 30°C to mid-logarithmic growth phase. Expression of *clpB* and *clpG* alleles was induced by IPTG addition. Total cell lysates were prepared and analyzed by SDS-PAGE and Coomassie staining. The positions of ClpB and ClpG are indicated. A protein marker is given. (c/d) *E. coli ΔclpB* cells harboring plasmids for expression of *E. coli clpB, clpB-K476C* or *P. aeruginosa clpG* (vc: empty vector control) were grown at 30°C to mid-logarithmic growth phase and shifted to 50°C. Serial dilutions of cells were prepared at the indicated time points, spotted on LB plates and incubated at 30°C (c). Colony numbers (CFU) were determined after 24 h and set to 100% for non-heat shocked samples (d). Standard deviations are provided (n=4). (e) *E. coli ΔclpB* cells harboring plasmids for expression of *E. coli clpB, clpB-K476C* or *P. aeruginosa clpG* (vc: empty vector control) were grown at 30°C to mid-logarithmic growth phase. Expression of *clpB* and *clpG* alleles was induced by IPTG addition. Total cell lysates were prepared and analyzed by SDS-PAGE and Coomassie staining. The positions of ClpB and ClpG are indicated. A protein marker is given.

Figure S2

(a) Binding of bis-ANS to Luciferase aggregates was determined. The denaturation conditions (temperature, Luciferase concentration) applied to generate Luciferase aggregates are indicated. The buffer control refers to bis-ANS fluorescence in absence of Luciferase aggregates. Bis-ANS fluorescence determined in presence of 200 nM aggregated Luciferase (42°C) was set as 100% and relative fluorescence of the other samples was calculated. Standard deviations are provided (n=3). (b/c) ClpG-mediated disaggregation of aggregated α-Glucosidase (b) or Malate Dehydrogenase (b) was determined in absence and presence of the DnaK system (KJE) by light scattering. Initial sample turbidity was set as 100%. Disaggregation rates (% turbidity/min) were determined and set as 100% for ClpG-mediated disaggregation. Standard deviations are provided (n=3).

Figure S3

(a) Aggregated Luciferase-YFP was incubated with ClpB, ClpB-K476C or ClpG. Disaggregation reactions with ClpB and ClpB-K476C included the cooperating DnaK system. YFP fluorescence spectra were recorded after 120 min incubation time. (b) Native Luciferase-YFP was incubated with ClpB, ClpB-K476C or ClpG. Samples with ClpB and ClpB-K476C included the cooperating DnaK system. Changes in YFP fluorescence were monitored. Initial YFP fluorescence was set as 100%.

Figure S4

E. coli ΔclpB cells harboring plasmids for IPTG-inducible expression of *E. coli clpB, clpB-K476C* or *P. aeruginosa clpG* were grown overnight at 30°C. Various dilutions were spotted on LB plates containing the indicated IPTG concentration and incubated at 30°C or 40°C for 24 h.
